# Healthcare utilization and costs among patients with non-functioning pituitary adenomas

**DOI:** 10.1007/s12020-019-01847-7

**Published:** 2019-03-22

**Authors:** Daniel J. Lobatto, Wilbert B. van den Hout, Amir H. Zamanipoor Najafabadi, Anath N. V. Steffens, Cornelie D. Andela, Alberto M. Pereira, Wilco C. Peul, Wouter R. van Furth, Nienke R. Biermasz, Thea P. M. Vliet Vlieland

**Affiliations:** 1 Department of Neurosurgery, Leiden University Medical Center, Leiden, The Netherlands; 2 Department of Medicine, Division of Endocrinology and Center for Endocrine Tumors, Leiden University Medical Center, Leiden, The Netherlands; 3 Medical Decision Making, Department of Biomedical Data Sciences, Leiden University Medical Center, Leiden, The Netherlands; 4 Department of Neurosurgery, Medical Center Haaglanden, The Hague, The Netherlands; 5 Department of Orthopaedics, Rehabilitation Medicine and Physical Therapy, Leiden University Medical Center, Leiden, The Netherlands

**Keywords:** Pituitary adenoma, NFPA, Healthcare utilization, Costs, Multidisciplinary, Value based healthcare

## Abstract

**Purpose:**

Non-functioning pituitary adenomas (NFPA) have a substantial impact on patients’ health status, yet research on the extent of healthcare utilization and costs among these patients is scarce. The objective was to determine healthcare usage, associated costs, and their determinants among patients treated for an NFPA.

**Methods:**

In a cross-sectional study, 167 patients treated for an NFPA completed four validated questionnaires. Annual healthcare utilization and associated costs were assessed through the medical consumption questionnaire (MTA iMCQ). In addition, the Leiden Bother and Needs Questionnaire for pituitary patients (LBNQ-Pituitary), Short Form-36 (SF-36), and EuroQol (EQ-5D) were administered. Furthermore, age, sex, endocrine status, treatment, and duration of follow-up were extracted from the medical records. Associations were analyzed using logistic/linear regression.

**Results:**

Annual healthcare utilization included: consultation of an endocrinologist (95% of patients), neurosurgeon (14%), and/or ophthalmologist (58%). Fourteen percent of patients had ≥1 hospitalization(s) and 11% ≥1 emergency room visit(s). Mean overall annual healthcare costs were € 3040 (SD 6498), highest expenditures included medication (31%), inpatient care (28%), and specialist care (17%). Factors associated with higher healthcare utilization and costs were greater self-perceived disease bother and need for support, worse mental and physical health status, younger age, and living alone.

**Conclusion:**

Healthcare usage and costs among patients treated for an NFPA are substantial and were associated with self-perceived health status, disease bother, and healthcare needs rather than endocrine status, treatment, or duration of follow-up. These findings suggest that targeted interventions addressing disease bother and unmet needs in the chronic phase are needed.

## Introduction

Patients with pituitary adenomas report impairments in health-related-quality of life (HRQoL) and a high disease burden [1–4]. In many cases, patients require lifelong (medical) treatment and monitoring by a multidisciplinary care team. Therefore, it is conceivable that total treatment costs are high, particularly in patients with endocrine deficits. Knowledge of the long-term healthcare utilization as well as the accompanying costs in patients with pituitary adenomas, however, is scarce.

NFPAs are highly prevalent among all pituitary adenomas [5] and can be considered as a separate entity, presenting with specific symptoms related to mass effects instead of hormonal excess. Studies describing healthcare utilization and/or costs of patients with pituitary adenomas, however, have focused primarily on functioning adenomas (i.e., Cushing’s disease, acromegaly, prolactinoma) [6–12]. The only study presenting data on healthcare use among patients with an NFPA lacked physician-specific information (e.g., specialties visited, number of visits), as well as factors associated with increased healthcare utilization or costs. The study did confirm higher healthcare utilization and costs compared to a reference population of people without a pituitary disease [11].

The current study aims to determine healthcare usage, associated costs and their determinants among patients treated for an NFPA. It was hypothesized that in patients with an NFPA hypopituitarism, postoperative radiotherapy, and/or shorter duration of follow-up were associated with higher healthcare utilization and costs. We anticipated an association between a higher disease burden and needs for support and higher healthcare utilization and costs. It is expected that the identification of these disease- or care-related determinants for healthcare utilization and costs will be helpful for the further understanding and improvement of healthcare utilization/cost drivers, as well as improve value for the patient by making care more efficient, and improve outcomes.

## Patients and methods

### Study design

We performed a cross-sectional study in the Leiden cohort consisting of patients treated for an NFPA. The Leiden University Medical Center (LUMC) is a tertiary referral center in the Netherlands for the treatment of pituitary adenomas. The study was approved by the ethical committee of the Leiden University Medical Center prior to the study (p12.067). This study was part of a larger project, also assessing work-related disability [13].

### Patients

All patients with an NFPA, aged ≥18 years, and currently under active follow-up, were identified from the hospital registries and invited by their treating physician by means of a letter to participate. Exclusion criteria were follow-up of <6 months, insufficient Dutch language skills, incapacity to fill out the questionnaires, and living abroad. Recruitment took place between September 2016 and March 2017. In case of no response, participants were re-approached once through a letter. Written consent was obtained from each participant after full explanation of the purpose and nature of the study.

### Assessments

The assessment consisted of a set of four validated questionnaires concerning healthcare usage and costs (Medical Consumption Questionnaire (iMTA MCQ)), perceived bother of disease and needs for support (Leiden Bother and Needs Questionnaire for pituitary patients (LBNQ-P)), HRQoL (Short Form-36 (SF-36), utility (EuroQol (EQ-5D)) and could be completed either digitally or on paper. In addition, sociodemographic and clinical data were collected from self-reports and medical records.

### Sociodemographic characteristics

The following disease-specific and sociodemographic characteristics were collected from the medical records: age, sex, date of diagnosis, and treatment. Self-reported characteristics were: marital status, educational level, employment status, and endocrine status. Level of education was categorized into low, intermediate or high, based on the guidelines of Statistics Netherlands (CBS) [14], which corresponds with UNESCO’s International Standard Classification of Education: Fields of Training and Education 2013 [15]. Employment status was categorized into three categories: (1) paid job, (2) no paid job, (3) retired. Treatment was divided into three categories: (1) wait-and-scan, (2) surgically treated patients, and (3) postoperative radiotherapy. Endocrine status was categorized as hypopituitarism (≥1 endocrine deficit(s)) or no deficits, according to hormone replacement therapy based on self-reported medication usage.

### Healthcare utilization

The iMTA MCQ assesses whether patients had an appointment with various healthcare professionals (HCPs) during the past 12 months and the frequency of appointments. For this study, those HCPs considered relevant for patients with a pituitary adenoma were included (e.g., endocrinologist, neurosurgeon, ophthalmologist, ENT-doctor, neurologist, radiation oncologist, cardiologist, internist). Patients were allowed to add additional HCPs through an open question (other). Furthermore, the questionnaire assesses home care (i.e., nursing care, (government-subsidized) household help, including frequency and duration), emergency care (i.e., ambulance rides, emergency room (ER) visits, including frequency), hospital admissions (including frequency and duration) and medication usage (including frequency and dosage). A binary specialist care utilization score was computed: specialist care utilization was defined as high or low according to the median total number of visits to medical specialists during the previous 12 months (high use: ≥4 visits).

### Perceived bother and needs for support

The LBNQ-Pituitary is a disease-specific questionnaire, which was developed based on focus group interviews with patients [16]. For this study, the LBNQ-Pituitary consisted of 26 items divided into five subscales: mood problems, negative illness perceptions, issues in sexual functioning, physical and cognitive complaints, and issues in social functioning, from which index scores can be calculated (range 0–100). A detailed description of how the items are scored has been previously published. Higher scores indicate greater bother by the consequences of the disease and higher needs for support [16].

### Health-Related-Quality of Life and utility

The *SF-36* is a 36-item HRQoL questionnaire, which covers eight domains: physical function, physical role, bodily pain, general health, vitality, social function, emotional role, and mental health. These subscales range from 0 to 100, from which the physical and mental component score can be calculated. Higher scores indicate better HRQoL [17].

The *EQ-5D* (5-level) is a utility questionnaire consisting of five domains: mobility, self-care, usual activities, pain/discomfort, and anxiety/depression, from which utility (range 0 to 1) can be calculated (EQ-5D index). The EQ-5D also includes a visual analog scale (VAS), which records self-reported health status (range 0–100). Higher scores indicate a better perceived health status [18].

### Costs

Cost prices were obtained according to the Dutch manual for costing research [19], and prices were based on reference prices for 2016. Relevant reference prices are presented in Supplementary Table 4. Conversion of costs can be made based on the purchasing power parity provided by the Organization for Economic Co-operation and Development (OECD), which was 0.816 per dollar in 2016 [https://data.oecd.org/conversion/purchasing-power-parities-ppp.htm, accessed on 2 November 2018].

### Statistics

Data entry was performed through an online survey platform. All statistical analyses were performed with IBM SPSS 23.0 software (IMB SPSS Inc., New York, USA). Continuous variables are presented as means and standard deviations (SD) or medians with interquartile ranges (IQR), analyzed through unpaired *t*-test or Mann–Whitney *U*, where applicable. Categorical variables were calculated as frequencies with percentages and comparisons were performed through Chi-square analyses and Fisher’s exact test, where applicable.

Logistic regression analysis was used to determine the relationship between specialist care utilization (high/low) as a dependent factor and possible contributing factors (i.e., disease-specific characteristics, sociodemographic characteristics, HRQoL, cost-utility, disease bother, needs for support). Associations are expressed as odds ratios (ORs) with the 95% confidence intervals (CIs) and *p*-values. Linear regression was used to determine the relationship between overall healthcare costs and all possible factors, which were in accordance with those of the logistic regression analysis. Associations are expressed as regression coefficients (B) with corresponding 95% confidence intervals and *p*-values.

To control for confounding, variables associated with both the determinant and the outcome and not in the causal pathway of the relationship of interest were used as covariates in the multivariate analyses [20]. All associations were corrected for age and gender, depending on the determinant also for treatment type. ANCOVA was performed for the analysis of the disease bother and needs for support, correcting for age and gender (Supplementary Table 5).

For all analyses, results were considered statistically significant if the *p*-value was smaller than 0.05 (two-sided). Missing data on the questionnaires were handled by complete case analysis due to the low amount of missings (<5%).

## Results

### Study population and patient characteristics

**Fig. 1:**
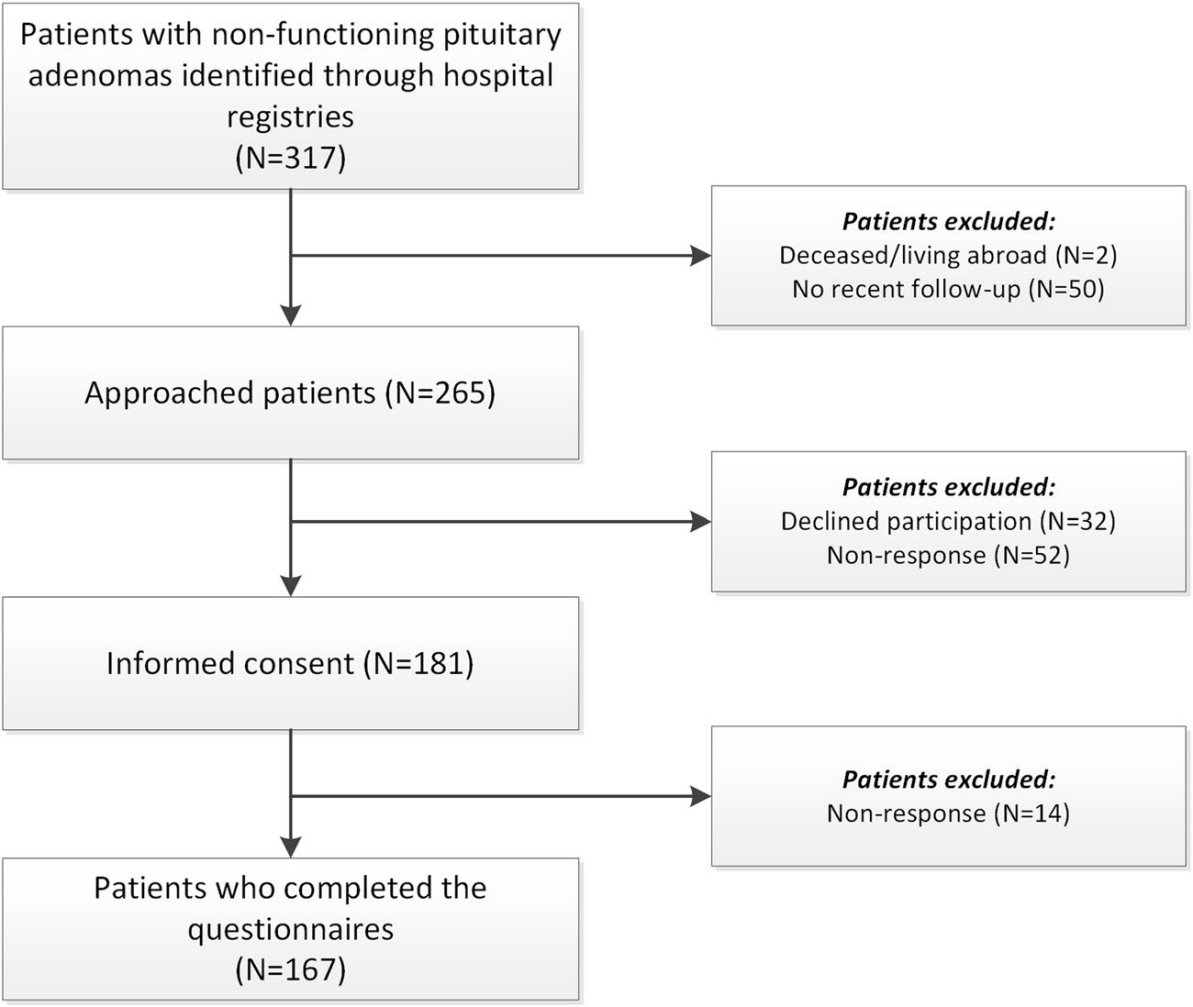
Flow chart of in-/exclusion of patients with an NFPA

A total of 317 patients with an NFPA were identified from the hospital registry. After exclusion of ineligible patients, letters were sent to 265 patients, ultimately enrolling 167 (63%) patients for this study (Fig. 1).

**Table 1:** Characteristics of 167 patients diagnosed with and treated for an NFPA categorized by endocrine deficits

	Total (*N* = 167)	No deficits (*N* = 46)	Hypopituitarism (*N* = 121)	*p*-value
Demographic characteristics				
Sex, *N* (%)				
Female	74 (44.3)	28 (60.9)	46 (38.0)	**0.008**
Age in years, mean (SD)	66.8 (12.1)	66.9 (11.2)	66.8 (12.5)	0.963
Marital status, *N* (%)				
Relationship/married	128 (76.6)	36 (77.8)	92 (76.0)	0.840
Education, *N* (%)				
Low	71 (42.5)	21 (45.7)	50 (41.3)	
Intermediate	41 (24.6)	10 (21.7)	31 (25.6)	
High	55 (32.9)	15 (32.6)	40 (33.1)	0.838
Employment status, *N* (%)				
Paid job	58 (34.9)	20 (44.4)	38 (31.4)	
No paid job	25 (15.1)	4 (8.9)	21 (17.4)	
Retired	84 (50.3)	22 (46.7)	62 (51.2)	0.229
Disease characteristics				
Time since diagnosis in years, median (IQR)	9.0 (4.8–18.4)	6.8 (4.5–13.5)	10.3 (5.1–19.4)	0.054
Treatment, *N* (%)				
Wait-and-scan	22 (13.2)	13 (28.3)	9 (7.4)	
Surgery	104 (62.3)	28 (60.9)	76 (62.8)	
Postoperative radiotherapy	41 (24.6)	5 (10.9)	36 (29.8)	<**0.001**
Current health status				
EQ-5D score, mean (SD)^a^	0.910 (0.089)	0.914 (0.075)	0.909 (0.094)	0.771
EQ-5D VAS, mean (SD)^a^	73.6 (20.5)	74.8 (21.8)	73.2 (20.1)	0.660
SF-36 PCS, mean (SD)^a^	44.5 (10.6)	44.6 (10.0)	44.4 (10.8)	0.899
SF-36 MCS, mean (SD)^a^	50.7 (10.3)	51.0 (10.7)	50.6 (10.2)	0.788
LBNQ-Pituitary index score, mean (SD)^b^	13.4 (15.9)	11.5 (15.6)	14.0 (16.0)	0.397

Due to rounding, not all percentages of the categorical variables add up to 100%

*NFPA* non-functioning pituitary adenoma, *N* number, *SD* standard deviation, *IQR* interquartile range, *VAS* visual analog scale, *EQ-5D* EuroQoL, *SF-36* short form-36, *LBNQ-Pituitary* Leiden bother and needs questionnaire-pituitary, *MCS* mental component scale, *PCS* physical component scale

^a^Higher scores indicate better HRQoL

^b^Lower scores indicate lower disease burden

Bold values indicates *p* < 0.05

**Fig. 2:**
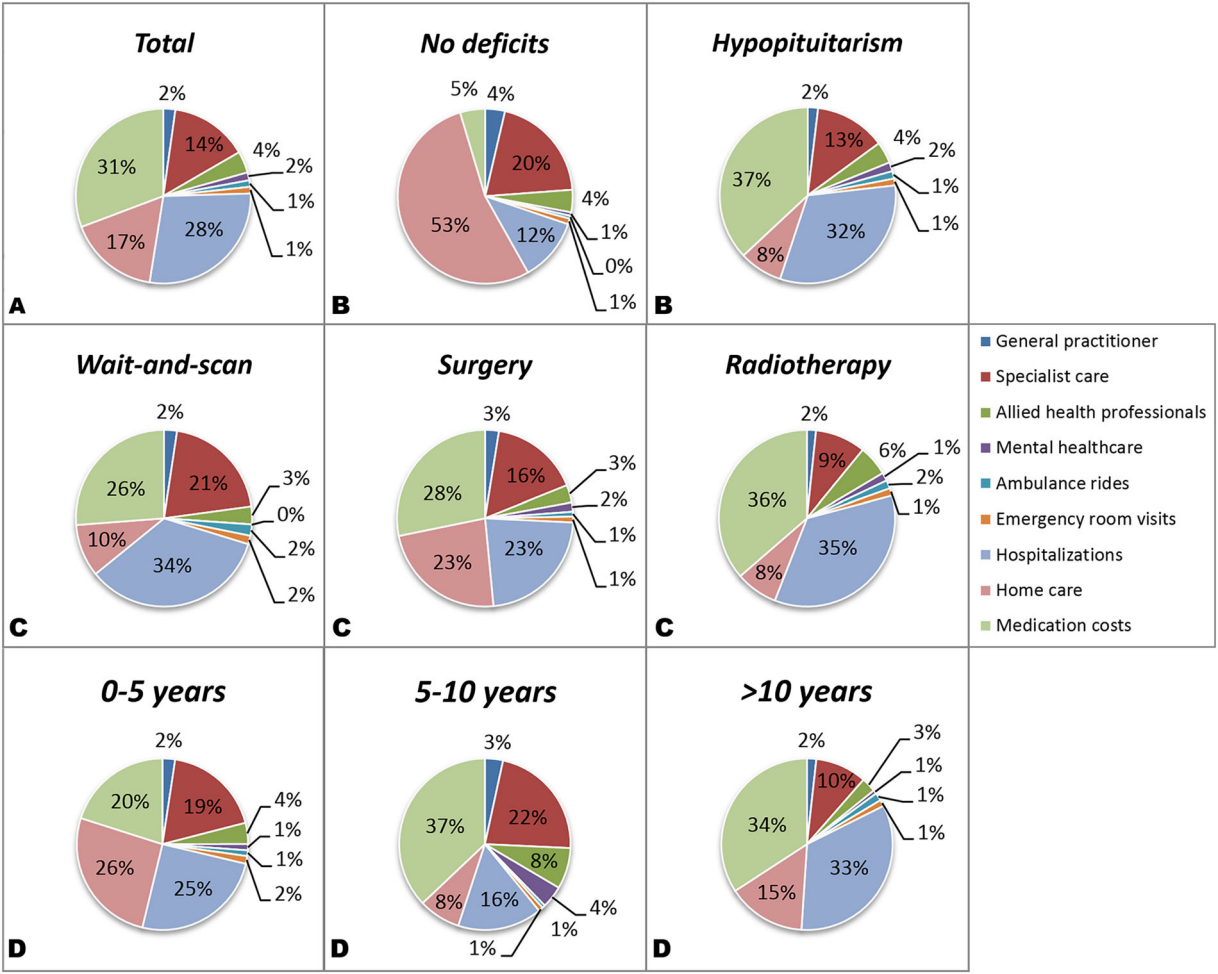
Overall costs among all patients with an NFPA **A**, categorized per endocrine status **B**, treatment algorithm **C** and duration of follow up **D**

In total, 93 (56%) patients were male, the mean age was 66.8 (SD 12.1) years and the median time since diagnosis was 9 years (IQR 4.8–18.4). Most patients (*n* = 105, 63%) had undergone surgical treatment, followed by postoperative radiotherapy (40, 24%), and only wait-and-scan approach (22, 13%). The majority of patients (121, 73%) had one or more endocrine deficits. Adrenal insufficiency was present in 77 patients (46%), which was highest among patients who had undergone postoperative radiotherapy (28, 68%), followed by surgical treatment (43, 41%) and wait-and-scan (6, 27%)(Table 1).

### Healthcare utilization

**Table 2:** Average healthcare usage over the past 12 months in 167 patients with NFPA categorized by endocrine deficits

Healthcare service	Total (*N* = 167)		No endocrine deficits (*N* = 46)		Hypopituitarism (*N* = 121)		
	Number of patients, %	Visits among those visiting, mean	Number of patients, %	Visits among those visiting, mean	Number of patients, %	Visits among those visiting, mean	*p*-value
General practitioner	51.5	4.1	50.0	4.8	52.1	3.9	0.101
NFPA related medical specialists							
Endocrinologist	94.6	2.1	89.1	1.8	96.7	2.2	0.685
Neurosurgeon	13.9	1.7	19.6	1.1	11.7	2.1	0.084
Ophthalmologist	58.4	2.1	65.2	2.4	55.8	2.0	0.311
ENT-doctor	9.0	1.8	15.2	1.1	6.7	2.4	0.179
Neurologist	9.6	2.2	8.7	2.0	10.0	2.3	0.885
Radiation oncologist	1.8	1.3	2.2	1.0	1.7	1.5	0.642
Cardiologist	10.2	1.8	10.9	1.2	9.9	2.0	0.907
Internist	11.4	2.2	10.9	2.0	11.7	2.2	0.151
Others	24.6	2.2	32.6	2.1	21.5	2.3	0.798
Total number of different specialists							
0	1.2	–	2.2	–	0.8	–	
1	24.6	1.9	23.9	1.5	24.8	2.0	
2	37.1	3.6	26.1	3.3	41.3	3.7	
3	20.4	5.3	26.1	4.8	18.3	5.5	
4 or more	16.2	11.9	21.7	10.4	14.2	12.8	0.300
Occupational care							
Occupational physician	6.6	3.8	6.5	1.0	6.7	4.9	0.054
Mental healthcare							
Psychologist/psychiatrist	8.4	8.2	4.3	5.0	10.0	8.8	0.764
Allied health professionals							
Physiotherapist	26.5	12.2	26.1	9.3	26.7	13.2	0.271
Speech therapist	0.6	10.0	–	–	0.8	10.0	1.00
Dietician	6.6	2.3	6.5	2.7	6.7	2.1	0.431
Occupational therapist	0	–	0	–	0	–	–
Total number of different allied health professionals							
0	64.7	–	69.6	–	63.3	–	
1	29.9	9.2	23.9	6.1	32.5	10.1	
2	4.2	17.4	4.3	14.0	4.2	18.8	
3	0.6	28.0	2.2	28.0	0	–	
4	0	–	0	–	0	–	0.244
Emergency care							
Ambulance rides, *N* (%), mean	6.0	1.2	2.2	1.0	7.4	1.2	1.00
Emergency room visit(s), *N* (%), mean	11.4	1.3	8.7	1.0	12.4	1.3	0.622
Hospital admission(s) *N* (%), duration	13.8	6.8	13.0	4.0	14.0	16.2	0.222
Home care							
Community nurse, *N* (%), hours	1.2	122.5	–	–	1.7	122.5	–
Informal care, *N* (%), hours	3.0	87.2	4.3	118.0	2.5	66.7	0.287
Household help, *N* (%), hours	3.6	132.3	6.5	185.3	2.5	79.3	0.306

*NFPA* non-functioning pituitary adenoma, *N* number, *SD* standard deviation

*p*-value based on number and frequency of visits, (bold) *p* < 0.05

### Primary care

The general practitioner was consulted in the previous year by 86 patients (52%). Fifty-eight patients (35%) had seen at least one other primary care health professional, most commonly the physiotherapist (44, 27%). There was no association between primary care utilization and patients with or without endocrine deficits, or duration of follow-up. Patients who had received postoperative radiotherapy had higher total physiotherapist and dietician visits (Tables 2 and 3).

### Specialist care

Nearly all patients (165, 99%) had visited a medical specialist in the previous year, with a median of 2 (IQR 1–3) specialists per year and a median of 3 visits (IQR 2–5) per year. The most commonly visited specialists were the endocrinologist (158 patients, 95%), the ophthalmologist (97, 58%), and the neurosurgeon (23, 14%) (Tables 2 and 3).

### Hospital admissions and emergency care

During the previous year, 23 patients (14%) had been admitted to a hospital at least once (mean hospital stay: 6.8; range 1–99 days). Furthermore, ten patients (6%) had at least one ambulance ride (mean 1.2; range 1–3 rides) and 19 patients (11%) had visited the ER at least once during the previous year (mean 1.3; range 1–5 visits). There were no significant differences in the amount of ambulance rides, ER visits, nor in the number or duration of hospitalizations between patients with or without endocrine deficits, based on applied treatments or duration of follow-up (Tables 2 and 3).

### Determinants for healthcare utilization

After correcting for relevant confounders, older patients (OR 0.973, 95% CI 0.948;1.000), patients with longer time since diagnosis (OR 0.966, 95% CI 0.933;1.000), as well as patients with a better mental and physical health status (SF-36) (OR 0.929, 95% CI 0.896;0.962), and higher utility (EQ-5D) (OR 0.913, 95% CI 0.870; 0.960) were significantly less likely to have high specialist care utilization. Contrarily, patients with higher overall perceived disease bother (OR 1.048, 95% CI 1.020;1.076) and needs for support (LBNQ-Pituitary) (OR 1.033, 95% CI 1.012;1.055) were significantly more likely to have high specialist care utilization. More specific, there was a significant need for support for issues regarding physical and cognitive complaints, mood, negative illness perceptions and social functioning, but not for sexual functioning. There were no differences in specialist care utilization between patients with postoperative radiotherapy compared to other treatment regimens, as well as for those with and without hypopituitarism (Table 4).

### Costs

The mean annual costs for patients with an NFPA were € 3040 (SD 6498) (Table 3). The three largest expenditures were (pituitary-specific) medication (31% of overall costs) inpatient care (28%), and specialist care (17%) (Fig. 2). The overall costs did not significantly differ between patients with and without hypopituitarism, even though there were significantly higher costs for medication among those with hypopituitarism, postoperative radiotherapy, and longer duration of follow-up (*p* < 0.05) (Table 3).

**Table 3:** Medical and medication costs in euros (€) over the past 12 months in 167 patients with an NFPA categorized by endocrine deficits

	Total (*N* = 167)				No endocrine deficits (*N* = 46)				Hypopituitarism (*N* = 121)				
Medical costs	Number of patients		Costs among those visiting		Number of patients		Costs among those visiting		Number of patients		Costs among those visiting		Overall *p*-value
	*N*	%	mean	SD	*N*	%	mean	SD	*N*	%	mean	SD	
General practitioner	86	51.5	135.67	131.28	23	50.0	159.00	156.99	63	52.1	126.97	120.68	0.498
Specialist care	165	98.8	444.52	511.96	45	97.8	438.82	428.71	120	99.2	446.66	541.52	0.878
Allied health professionals^a^	58	34.9	348.21	378.54	14	30.4	289.93	245.31	44	36.7	366.75	412.67	0.339
Mental healthcare^b^	14	8.4	525.71	449.88	2	4.3	320.00	271.53	12	10.0	560.00	472.77	0.211
Ambulance rides	10	6.0	618.00	325.71	1	2.2	515.00	–	9	7.4	629.44	343.33	0.215
Emergency room visits	19	11.4	327.16	169.22	4	8.7	259.00	0.00	15	12.4	345.33	187.45	0.323
Hospitalization	23	13.8	6188.00	10737.13	6	13.0	1904.00	2624.48	17	14.0	7700.00	12126.60	0.282
Home care^c^	7	4.2	12094.57	5731.49	3	6.5	17462.67	2058.55	4	3.3	8068.50	3527.91	0.060
Total medical costs	167	100	2103.43	6420.38	46	100	2028.41	4610.01	121	100	2131.95	7003.58	0.926

Medication costs	Number of patients		Costs among those using medication		Number of patients		Costs among those using medication		Number of patients		Costs among those using medication		Overall *p*-value
	*N*	%	Mean	SD	*N*	%	Mean	SD	*N*	%	Mean	SD	
Androgel	56	33.5	445.88	258.61	0	–	–	–	56	46.3	445.88	258.61	<**0.001**
Desmopressine	11	6.6	99.12	66.92	0	–	–	–	11	9.0	99.12	66.92	0.079
Thyrax	90	53.9	34.29	12.94	0	–	–	–	90	74.4	34.29	12.94	<**0.001**
Genotropin	30	18.0	2917.47	1505.46	0	–	–	–	30	24.8	2917.47	1505.46	**0.001**
Cabergoline	3	1.8	1887.71	2227.07	1	2.2	4423.92	–	2	1.7	619.60	520.66	0.158
Quinagolide	2	1.2	1056.00	1405.73	1	2.2	62.00	–	1	0.8	2050.00	–	0.572
Hydrocortison	77	46.1	411.82	218.94	0	–	–	–	77	63.6	411.82	218.94	<**0.001**
Anticonceptives	4	2.4	43.41	31.03	2	4.3	27.90	–	2	1.7	58.93	43.88	0.861
Total drug costs	125	74.9	1250.63	1610.18	4	8.7	1135.43	2192.39	121	100	1254.44	1599.53	<**0.001**
Overall costs	167	100	3039.53	6498.22	46	100	2127.15	4632.22	121	100	3386.39	7065.90	0.265

Reference prices are presented in Supplementary Table 4

*NFPA* non-functioning pituitary adenoma, *N* number, *IQR* interquartile range

^a^Physiotherapists, Speech therapists, Dieticians, Occupational therapists

^b^Psychiatrists, psychologists

^c^Community nurse, informal care, household help

Bold values indicates *p* < 0.05

### Determinants for increased costs

Concerning healthcare costs, patients who were living alone had significantly higher healthcare costs (B 2960, 95% CI 510;5415) compared to those in a relationship. Patients with worse mental (B −107, 95% CI −206;−9) or worse physical health status (SF-36) (B −178, 95% CI −273;−82), lower utility (EQ-5D) (B −267, 95% CI −374;−161), greater disease bother (B 123, 95% CI 58;188), and a higher need for support (LBNQ-Pituitary) (B 130, 95% CI 79;180) also had significantly higher costs, which was the case for all domains. Hypopituitarism, postoperative radiotherapy and duration of follow-up were not associated with higher costs (Table 4).

**Table 4:** Logistic/linear regression analysis per determinant for medical specialist utilization and costs among patients with an NFPA

Determinant	High specialist utilization (adjusted for demographics)			Healthcare costs (adjusted for demographics)		
	OR	95% CI	*p*-value	*B*	95% CI	*p*-value
Sociodemographic						
Sex (ref: male gender)^a^	1.504	0.804;2.814	0.202	1606	−404;3617	0.117
Age^b^	**0.973**	**0.948;1.000**	**0.047**	51	−32;133	0.226
Marital status (ref: relationship/married)^a,b^						
Single/divorced/widow	2.195	0.982;4.905	0.055	**2963**	**510;5415**	**0.018**
Education (ref: high)^a,b^						
Intermediate	1.559	0.728;3.337	0.253	1666	−980;4311	0.216
Low	1.778	0.768;4.116	0.179	687	−1712;3087	0.572
Employment status (ref: paid job)^a,b^						
No paid job	0.580	0.206;1.635	0.303	−941	−4235;2354	0.574
Retired	0.608	0.221;1.674	0.336	−1061	−4293;2171	0.518
Disease specific						
Time since diagnosis^a,b^	**0.966**	**0.933;1.000**	**0.047**	91	−9;191	0.074
Treatment (ref: wait-and-scan)^a,b^						
Surgery	0.656	0.483;3.179	0.656	484	−2520;3488	0.751
Postoperative radiotherapy	0.644	0.442;3.743	0.644	1895	−1520;5310	0.275
Endocrine status (ref: no deficits)^a,b,c^						
Hypopituitarism	0.734	0.414;1.861	0.734	1456	−945;3857	0.233
HRQoL, utility, disease bother and needs for support						
SF-36^a,b^						
Mental component scale	**0.942**	**0.909;0.976**	**0.001**	−**107**	−**206;**−**9**	**0.033**
Physical component scale	**0.929**	**0.896;0.962**	**<0.001**	−**178**	−**273;**−**82**	<**0.001**
EQ-5D^a,b^						
EQ index (rescaled to 0–100)	**0.913**	**0.870;0.960**	**<0.001**	−**267**	−**374;**−**161**	<**0.001**
EQ VAS (scale 0–100)	**0.968**	**0.951;0.986**	**0.001**	−**98**	−**146;**−**51**	<**0.001**
Disease bother (LBNQ-Pituitary)^a,b^						
Physical and cognitive complaints	**1.037**	**1.016;1.059**	<**0.001**	**77**	**25;130**	**0.004**
Mood	**1.036**	**1.015;1.057**	**0.001**	**60**	**7;114**	**0.028**
Negative illness perceptions	**1.044**	**1.018;1.070**	**0.001**	61	−5;127	0.070
Sexual functioning	**1.021**	**1.003;1.038**	**0.018**	**111**	**64;159**	<**0.001**
Social functioning	**1.030**	**1.004;1.056**	**0.021**	**146**	**86;207**	<**0.001**
Total index score	**1.048**	**1.020;1.076**	**0.001**	**123**	**58;188**	<**0.001**
Needs for support (LBNQ-Pituitary)^a,b^						
Physical and cognitive complaints	**1.031**	**1.013;1.049**	**0.001**	**88**	**44;131**	<**0.001**
Mood	**1.026**	**1.010;1.043**	**0.001**	**80**	**38;122**	<**0.001**
Negative illness perceptions	**1.019**	**1.004;1.035**	**0.013**	**51**	**8;95**	**0.021**
Sexual functioning	1.013	0.998;1.029	0.096	**131**	**90;172**	<**0.001**
Social functioning	**1.023**	**1.001;1.044**	**0.036**	**155**	**104;206**	<**0.001**
Total index score	**1.033**	**1.012;1.055**	**0.002**	**130**	**79;180**	<**0001**

SF-36, EQ-5D: higher scores indicate better HRQoL or utility/LBNQ-Pituitary: lower scores indicate lower disease bother or needs for support

*Ref* reference category, (bold) *p* < 0.05, *OR* odds ratio, *CI* confidence interval, *HRQoL* Health-Related-Quality of Life, *SF-36* Short Form-36, *EQ-5D* EuroQoL, *LBNQ-Pituitary* Leiden Bother and Needs Questionnaire

^a,b,c^Adjusted for age (1), gender (2), treatment (3)

## Discussion

The present study demonstrated that the overall healthcare utilization and costs in patients with an NFPA are substantial. Furthermore, this study shows that the endocrinologist and ophthalmologist are both actively involved in the care of over 50% of patients with an NFPA. In contrast to our hypothesis, overall healthcare utilization and overall costs did not differ between patients with or without endocrine deficits, or between the various treatments. Also, longer duration of follow-up was associated with lower healthcare utilization instead of higher utilization. These findings are intriguing, especially since it was anticipated that the burden of multiple hormone replacement therapy would have a significant impact on overall healthcare utilization.

Differences between patients appeared to be more related to subjective measurements such as HRQoL, disease bother and needs for support than objective outcomes or treatment variation. For instance, there was a strong association between lower HRQoL, higher self-perceived disease bother (on all domains of the LBNQ-pituitary) and needs for support (on all domains, except for sexual functioning) and increased healthcare utilization and costs. This makes patient-reported outcome measures (PROMS) a promising tool to gain better insight into the patient’s condition and when to consider interventions to reduce healthcare utilization and costs and to optimize care trajectories. Further investigation towards optimal strategies supporting this hypothesis is necessary, perhaps through self-management interventions [21, 22].

Our study can be best compared to the study by Swearingen et al. [11], which is the only other currently available study reporting on healthcare utilization among 3792 patients with an NFPA. Comparable results were found for hospitalizations and office visits, however they found a higher number of ER visits (24% vs. 11%). This study, however, presented aggregated data from insurance claims databases, limiting comprehensive insight into which healthcare providers are consulted, lacked information on treatment and endocrine status and did not look at determinants for healthcare utilization and costs.

Other studies among patients with a functioning pituitary adenoma have mostly shown higher rates of hospitalizations (range 9–38.4% vs. 14%), comparable rates of proportion of patients visiting specialists (range 94–99% vs. 99%), and also higher ER visits (23–34% vs. 11%) [6–12]. The major differences in disease characteristics, however, limit comparability.

Concerning the costs of patients treated for an NFPA, the mean total costs found in our study were approximately fourfold lower compared to those reported by Swearingen et al. [11] ($ 13,708 vs. € 3039), which can be explained, at least in part, by the higher healthcare costs in the USA [23], but also show the variation between costs for patients with different types of pituitary adenomas. The most notable difference is the mean costs for medication, which is nearly ten times as high among the study by Swearingen et al. ($ 11,181 vs. € 1250). With regard to functioning adenomas, the mean total costs among patients with Cushing’s disease ranged between $ 26,269 and $ 34,992; for acromegaly between € 9200 and $ 32,807 [6–12]. Both are considerably higher compared to the costs found in our study.

Pertaining to determinants, to the best of our knowledge, no other study has described determinants for healthcare utilization or costs of care for patients with an NFPA. One other study among patients with acromegaly previously reported that younger age, female gender, and hypopituitarism were associated with higher healthcare costs, and that the presence of an increasing amount of comorbidities was associated with an increased risk for hospitalizations and ER visits [7]. These results could not be confirmed in our study.

A strength of our study was the high response rate (63%). The use of self-reported information on healthcare services was another strength of our study. This has recently been reported as the most suitable method for the measurement of healthcare utilization [24], thereby supporting the results presented here. This, however, was also a limitation of our study; since questionnaires were based on self-reports, it is possible that patients had difficulty distinguishing between various medical terms, i.e., differentiating between radiologists and radiation oncologists. Another important limitation is that even though we acknowledge that comorbidities are an important factor for a patient’s HRQoL [7, 25], we were unable to analyze the impact of comorbidities in our study. Furthermore, the decision to invite only those patients who had visited the outpatient clinic in the prior 2 years (based on the tertiary referral function of our center) may have introduced a selection bias. We anticipated that this would influence results in both a negative and a positive way, since not only patients with better health status are referred back to the center they were referred by, but also patients with worse health status who are unable to travel to our center. The single center setting in which this study took place is another limitation that restricts generalizability of this study. However, by providing mean visits per patient, comparisons between healthcare systems can be made. Finally, we only included pituitary-specific medication in the analysis of the medication costs, which underestimates total medication costs.

The high active involvement by the endocrinologist and ophthalmologist in the care of patients with an NFPA in combination with the association between subjective determinants for healthcare utilization and costs are potential targets for future interventions. A next step could be to define trajectories of care and match these with the health status and healthcare needs of specific subgroups of patients in order to generate patient-tailored care. This might ultimately improve HRQoL and could lead to cost reductions in the long haul, however, prospective studies are necessary to confirm this hypothesis.

## Conclusion

Healthcare utilization and costs among patients with an NFPA are substantial. Intriguingly, the extent of healthcare utilization and costs is independent of endocrine status and treatment algorithm, and costs are independent of duration of follow-up. Instead, worse HRQoL and more bother by the negative consequences of the disease and needs for support were associated with higher healthcare utilization and costs and can potentially be used as a tool to differentiate healthcare usage and cost drivers.

## Supplementary information

Supplementary Table 1a

Supplementary Table 1b

Supplementary Table 2a

Supplementary Table 2b

Supplementary Table 3a

Supplementary Table 3b

Supplementary Table 4

Supplementary Table 5a

Supplementary Table 5b

Supplementary Table 5c
