# Supplementary material for: Healthcare utilization and costs among patients with non-functioning pituitary adenomas
Source: Endocrine. 2019 Mar 22;64(2):330–40. doi: 10.1007/s12020-019-01847-7 (PMC6531397; doi:10.1007/s12020-019-01847-7)
Supplement: Supplementary file 1 — Supplementary Table 1a [file 12020_2019_1847_MOESM1_ESM.docx]

| **Supplementary table 1a.** Characteristics of 167 patients diagnosed with and treated for NFPA categorized by treatment | | | | | | | | | | |
| --- | --- | --- | --- | --- | --- | --- | --- | --- | --- | --- |
|  | **Total**  **(N=167)** | | | **Wait-and-scan**  **(N=22)** | | **Surgery**  **(N=104)** | | **Postoperative**  **Radiotherapy**  **(N=41)** | | **p-value** |
| **Demographic characteristics** |  |  |  | |  |  |  |  |  |  |
| Sex, N (%) |  |  |  | |  |  |  |  |  |  |
| Female | 74 | (44.3) | 8 | | (36.4) | 44 | (42.3) | 22 | (53.7) | .336 |
| Age in years, mean (SD) | 66.8 | (12.1) | 68.7 | | (12.6) | 67.6 | (12.5) | 63.9 | (10.5) | .197 |
| Marital status, N (%) |  |  |  | |  |  |  |  |  |  |
| Relationship/married | 128 | (76.6) | 17 | | (77.3) | 83 | (79.8) | 28 | (68.3) | .336 |
| Education, N (%) |  |  |  | |  |  |  |  |  |  |
| Low | 71 | (42.5) | 6 | | (27.3) | 45 | (43.3) | 20 | (48.8) |  |
| Intermediate | 41 | (24.6) | 6 | | (27.3) | 25 | (24.0) | 10 | (24.4) |  |
| High | 55 | (32.9) | 10 | | (45.5) | 34 | (32.7) | 11 | (26.8) | .527 |
| Employment status, N (%) |  |  |  | |  |  |  |  |  |  |
| Paid job | 58 | (34.9) | 10 | | (45.5) | 38 | (36.9) | 10 | (24.4) |  |
| No paid job | 25 | (15.1) | 0 | | (0.0) | 12 | (11.7) | 13 | (31.7) |  |
| Retired | 84 | (50.3) | 12 | | (54.5) | 54 | (51.9) | 18 | (43.9) | **.008** |
| **Disease characteristics** |  |  |  | |  |  |  |  |  |  |
| Time since diagnosis in years, median (IQR) | 9.0 | (4.8-18.4) | 10.1 | | (6.7-16.0) | 6.8 | (4.1-13.3) | 18.4 | (11.1-24.9) | **<.001** |
| Endocrine status, N (%) |  |  |  | |  |  |  |  |  |  |
| Hypopituitarism | 121 | (72.5) | 9 | | (40.9) | 76 | (73.1) | 36 | (87.8) | **<.001** |
| **Current Health Status** |  |  |  | |  |  |  |  |  |  |
| EQ-5D score, mean (SD)* | 0.910 | (0.089) | 0.917 | | (0.068) | 0.911 | (0.085) | 0.906 | (0.110) | .894 |
| EQ-5D VAS, mean (SD)* | 73.6 | (20.5) | 72.7 | | (14.4) | 75.1 | (21.1) | 70.5 | (21.6) | .479 |
| SF-36 PCS, mean (SD)* | 44.5 | (10.6) | 45.8 | | (9.4) | 45.1 | (10.9) | 42.1 | (10.1) | .242 |
| SF-36 MCS, mean (SD)* | 50.7 | (10.3) | 51.0 | | (10.4) | 50.5 | (10.7) | 50.9 | (9.5) | .965 |
| LBNQ-Pituitary index score, mean (SD)† | 13.4 | (15.9) | 11.3 | | (15.4) | 11.9 | (15.7) | 18.1 | (15.9) | .092 |
| NFPA (non-functioning pituitary adenoma), N (number), SD (standard deviation), IQR (interquartile range), VAS (visual analogue scale), EQ-5D (EuroQoL), SF-36 (short form-36), LBNQ-Pituitary (Leiden bother and needs questionnaire-pituitary), MCS (mental component scale), PCS (physical component scale), (bold) p < 0.05  * Higher scores indicate better HRQoL  † Lower scores indicate lower disease burden  Due to rounding, not all percentages of the categorical variables add up to 100% | | | | | | | | | | |
