# Supplementary material for: Healthcare utilization and costs among patients with non-functioning pituitary adenomas
Source: Endocrine. 2019 Mar 22;64(2):330–40. doi: 10.1007/s12020-019-01847-7 (PMC6531397; doi:10.1007/s12020-019-01847-7)
Supplement: Supplementary file 2 — Supplementary Table 1b [file 12020_2019_1847_MOESM2_ESM.docx]

| **Supplementary table 1b.** Characteristics of 167 patients diagnosed with and treated for NFPA categorized by duration of follow-up | | | | | | | | | |
| --- | --- | --- | --- | --- | --- | --- | --- | --- | --- |
|  | **Total**  **(N=167)** | | **0-5 years**  **(N=43)** | | **5-10 years**  **(N=45)** | | **>10 years**  **(N=79)** | | **p-value** |
| **Demographic characteristics** |  |  |  |  |  |  |  |  |  |
| Sex, N (%) |  |  |  |  |  |  |  |  |  |
| Female | 74 | (44.3) | 17 | (39.5) | 20 | (44.4) | 37 | (46.8) | .740 |
| Age in years, mean (SD) | 66.8 | (12.1) | 63.9 | (13.2) | 64.0 | (11.7) | 70.1 | (11.0) | **.004** |
| Marital status, N (%) |  |  |  |  |  |  |  |  |  |
| Relationship/married | 128 | (76.6) | 34 | (79.1) | 38 | (84.4) | 56 | (70.9) | .209 |
| Education, N (%) |  |  |  |  |  |  |  |  |  |
| Low | 71 | (42.5) | 14 | (32.6) | 24 | (53.3) | 33 | (41.8) |  |
| Intermediate | 41 | (24.6) | 14 | (32.6) | 9 | (20.0) | 18 | (22.8) |  |
| High | 55 | (32.9) | 15 | (34.9) | 12 | (26.7) | 28 | (35.4) | .332 |
| Employment status, N (%) |  |  |  |  |  |  |  |  |  |
| Paid job | 58 | (34.9) | 25 | (58.1) | 14 | (31.1) | 19 | (24.4) |  |
| No paid job | 25 | (15.1) | 2 | (4.7) | 10 | (22.2) | 13 | (16.7) |  |
| Retired | 84 | (50.3) | 16 | (37.2) | 21 | (46.7) | 47 | (59.5) | .002 |
| **Disease characteristics** |  |  |  |  |  |  |  |  |  |
| Time since diagnosis in years, median (IQR) | 9.0 | (4.8-18.4) | 3.1 | (2.5-4.3) | 6.9 | (6.2-8.0) | 18.7 | (13.5-24.9) | **<.001** |
| Treatment, N (%) |  |  |  |  |  |  |  |  |  |
| Wait-and-scan | 22 | (13.2) | 4 | (9.3) | 6 | (13.3) | 12 | (15.2) |  |
| Surgery | 104 | (62.3) | 38 | (88.4) | 31 | (68.9) | 35 | (44.3) |  |
| Postoperative radiotherapy | 41 | (24.6) | 1 | (2.3) | 8 | (17.8) | 32 | (40.5) | **<.001** |
| Endocrine status, N (%) |  |  |  |  |  |  |  |  |  |
| Hypopituitarism | 121 | (72.5) | 30 | (69.8) | 28 | (62.2) | 63 | (79.7) | .099 |
| **Current Health Status** |  |  |  |  |  |  |  |  |  |
| EQ-5D score, mean (SD)* | 0.910 | (0.089) | 0.926 | (0.067) | 0.913 | (0.079) | 0.900 | (0.103) | .316 |
| EQ-5D VAS, mean (SD)* | 73.6 | (20.5) | 74.9 | (21.8) | 75.3 | (17.8) | 72.0 | (21.4) | .634 |
| SF-36 PCS, mean (SD)* | 44.5 | (10.6) | 46.1 | (10.0) | 44.3 | (11.6) | 43.6 | (10.3) | .468 |
| SF-36 MCS, mean (SD)* | 50.7 | (10.3) | 50.2 | (11.3) | 48.1 | (11.8) | 52.4 | (8.4) | .073 |
| LBNQ-Pituitary index score, mean (SD)† | 13.4 | (15.9) | 11.8 | (16.7) | 17.9 | (18.2) | 11.4 | (13.3) | .076 |
| NFPA (non-functioning pituitary adenoma), N (number), SD (standard deviation), IQR (interquartile range), VAS (visual analogue scale), EQ-5D (EuroQoL), SF-36 (short form-36), LBNQ-Pituitary (Leiden bother and needs questionnaire-pituitary), MCS (mental component scale), PCS (physical component scale), (bold) p < 0.05  * Higher scores indicate better HRQoL  † Lower scores indicate lower disease burden  Due to rounding, not all percentages of the categorical variables add up to 100% | | | | | | | | | |
