# Supplementary material for: Healthcare utilization and costs among patients with non-functioning pituitary adenomas
Source: Endocrine. 2019 Mar 22;64(2):330–40. doi: 10.1007/s12020-019-01847-7 (PMC6531397; doi:10.1007/s12020-019-01847-7)
Supplement: Supplementary file 3 — Supplementary Table 2a [file 12020_2019_1847_MOESM3_ESM.docx]

| **Supplementary table 2a.** Average healthcare usage over the past 12 months in 167 patients with an NFPA categorized by treatment | | | | | | | | | |
| --- | --- | --- | --- | --- | --- | --- | --- | --- | --- |
| **Healthcare service** | **Total**  **(N=167)** | | **Wait-and-scan (N=22)** | | **Surgery**  **(N=104)** | | **Postoperative radiotherapy**  **(N=41)** | |  |
|  | Number of patients, % | Visits among those visiting, mean | Number of patients, % | Visits among those visiting, mean | Number of patients, % | Visits among those visiting, mean | Number of patients, % | Visits among those visiting, mean | p-value |
| General practitioner | 51.5 | 4.1 | 40.9 | 4.0 | 51.0 | 4.0 | 46.3 | 4.4 | .950 |
| **NFPA related medical specialists** |  |  |  |  |  |  |  |  |  |
| Endocrinologist | 94.6 | 2.1 | 86.4 | 1.9 | 94.2 | 2.2 | 100.0 | 1.8 | .560 |
| Neurosurgeon | 13.9 | 1.7 | 0 | - | 20.2 | 1.7 | 4.9 | 2.0 | .039 |
| Ophthalmologist | 58.4 | 2.1 | 72.7 | 1.7 | 57.7 | 2.2 | 48.8 | 2.4 | .904 |
| ENT-doctor | 9.0 | 1.8 | 9.1 | 1.5 | 9.6 | 1.8 | 7.3 | 2.0 | .964 |
| Neurologist | 9.6 | 2.2 | 0 | - | 8.7 | 2.2 | 17.1 | 2.1 | .193 |
| Radiation oncologist | 1.8 | 1.3 | 0 | - | 1.0 | 2.0 | 4.9 | 1.0 | .146 |
| Cardiologist | 10.2 | 1.8 | 18.2 | 2.5 | 9.6 | 1.6 | 7.3 | 1.3 | .166 |
| Internist | 11.4 | 2.2 | 9.1 | 1.0 | 13.5 | 2.5 | 4.9 | 1.0 | .195 |
| Others | 24.6 | 2.2 | 36.4 | 3.5 | 20.2 | 2.1 | 29.3 | 1.4 | .064 |
| Total number of different specialists |  |  |  |  |  |  |  |  |  |
| 0 | 1.2 | - | 4.5 | - | 1.0 | - | 0 | - |  |
| 1 | 24.6 | 1.9 | 13.6 | 1.7 | 27.2 | 1.8 | 24.4 | 2.1 |  |
| 2 | 37.1 | 3.6 | 36.4 | 3.1 | 35.0 | 3.9 | 43.9 | 3.3 |  |
| 3 | 20.4 | 5.3 | 22.7 | 5.2 | 20.4 | 5.1 | 19.5 | 5.9 |  |
| 4 or more | 16.2 | 11.9 | 22.7 | 11.2 | 16.3 | 12.9 | 12.2 | 9.2 | .689 |
| **Occupational care** |  |  |  |  |  |  |  |  |  |
| Occupational physician | 6.6 | 3.8 | 9.1 | 2.0 | 4.8 | 4.8 | 9.8 | 3.5 | .867 |
| **Mental healthcare** |  |  |  |  |  |  |  |  |  |
| Psychologist/psychiatrist | 8.4 | 8.2 | 0 | - | 7.7 | 9.4 | 14.6 | 6.7 | .468 |
| **Allied health professionals** |  |  |  |  |  |  |  |  |  |
| Physiotherapist | 26.5 | 12.2 | 27.3 | 7.5 | 22.1 | 10.7 | 36.6 | 16.3 | **.044** |
| Speech therapist | 0.6 | 10.0 | 0 | - | 0 | - | 2.4 | 10.0 | .219 |
| Dietician | 6.6 | 2.3 | 0 | - | 3.8 | 2.0 | 17.1 | 2.4 | **.006** |
| Occupational therapist | 0 | - | 0 | - | 0 | - | 0 | - | - |
| Total number of different allied health professionals |  |  |  |  |  |  |  |  |  |
| 0 | 64.7 | - | 68.2 | - | 70.9 | - | 48.8 | - |  |
| 1 | 29.9 | 9.2 | 27.3 | 6.3 | 27.2 | 8.7 | 39.0 | 11.3 |  |
| 2 | 4.2 | 17.4 | 4.5 | 11.0 | 1.9 | 17.0 | 9.8 | 19.3 |  |
| 3 | 0.6 | 28.0 | 0 | - | 0 | - | 2.4 | 28.0 |  |
| 4 | 0 | - | 0 | - | 0 | - | 0 | - | .089 |
| **Emergency care** |  |  |  |  |  |  |  |  |  |
| Ambulance rides, N(%), mean | 6.0 | 1.2 | 9.1 | 1.0 | 4.8 | 1.0 | 7.3 | 1.7 | .350 |
| Emergency room visit(s), N(%), mean | 11.4 | 1.3 | 13.6 | 1.0 | 9.6 | 1.2 | 14.6 | 1.5 | .531 |
| Hospital admission(s) N(%), duration | 13.8 | 6.8 | 22.7 | 7.2 | 13.5 | 5.1 | 9.8 | 12.5 | .205 |
| **Home care** |  |  |  |  |  |  |  |  |  |
| Community nurse, N(%), hours | 1.2 | 122.5 | 0 | - | 1.0 | 20.0 | 2.4 | 225.0 | - |
| Informal care, N(%), hours | 3.0 | 87.2 | 4.5 | 96.0 | 2.9 | 85.3 | 2.4 | 84.0 | .990 |
| Household help, N(%), hours | 3.6 | 132.3 | 0 | - | 4.9 | 146.8 | 2.4 | 60.0 | .234 |
| NFPA (non-functioning pituitary adenoma), N (number), SD (standard deviation)  p-value based on number and frequency of visits, (bold) p < 0.05 | | | | | | | | | |
