# Supplementary material for: Healthcare utilization and costs among patients with non-functioning pituitary adenomas
Source: Endocrine. 2019 Mar 22;64(2):330–40. doi: 10.1007/s12020-019-01847-7 (PMC6531397; doi:10.1007/s12020-019-01847-7)
Supplement: Supplementary file 4 — Supplementary Table 2b [file 12020_2019_1847_MOESM4_ESM.docx]

| **Supplementary table 2b.** Average healthcare usage over the past 12 months in 167 patients with an NFPA categorized by follow-up | | | | | | | | | |
| --- | --- | --- | --- | --- | --- | --- | --- | --- | --- |
| **Healthcare service** | **Total**  **(N=167)** | | **0-5 years**  **(N=43)** | | **5-10 years**  **(N=45)** | | **>10 years**  **(N=79)** | |  |
|  | Number of patients, % | Visits among those visiting, mean | Number of patients, % | Visits among those visiting, mean | Number of patients, % | Visits among those visiting, mean | Number of patients, % | Visits among those visiting, mean | p-value |
| General practitioner | 51.5 | 4.1 | 55.8 | 4.3 | 40.0 | 5.1 | 49.4 | 3.6 | .411 |
| **NFPA related medical specialists** |  |  |  |  |  |  |  |  |  |
| Endocrinologist | 94.6 | 2.1 | 90.7 | 2.9 | 97.8 | 2.1 | 94.9 | 1.6 | **.018** |
| Neurosurgeon | 13.9 | 1.7 | 23.3 | 1.4 | 22.2 | 2.0 | 3.8 | 1.7 | **.008** |
| Ophthalmologist | 58.4 | 2.1 | 62.8 | 2.6 | 64.4 | 2.0 | 50.6 | 2.0 | .127 |
| ENT-doctor | 9.0 | 1.8 | 7.0 | 3.3 | 6.7 | 1.0 | 11.4 | 1.6 | .558 |
| Neurologist | 9.6 | 2.2 | 14.0 | 2.8 | 8.9 | 2.0 | 7.6 | 1.7 | .184 |
| Radiation oncologist | 1.8 | 2.7 | 4.7 | 1.5 | 2.2 | 1.0 | 0 | - | .315 |
| Cardiologist | 10.2 | 1.8 | 9.3 | 2.5 | 8.9 | 1.0 | 11.4 | 1.8 | .623 |
| Internist | 11.4 | 2.2 | 11.6 | 3.6 | 11.1 | 1.4 | 10.1 | 1.8 | .357 |
| Others | 24.6 | 2.2 | 27.9 | 2.8 | 20.0 | 2.2 | 25.3 | 1.8 | .167 |
| Total number of different specialists |  |  |  |  |  |  |  |  |  |
| 0 | 1.2 | - | 2.3 | - | 0 | - | 1.3 | - |  |
| 1 | 24.6 | 1.9 | 20.9 | 2.8 | 15.6 | 1.6 | 31.6 | 1.6 |  |
| 2 | 37.1 | 3.6 | 32.6 | 3.2 | 42.2 | 4.2 | 36.7 | 3.5 |  |
| 3 | 20.4 | 5.3 | 25.6 | 6.9 | 22.2 | 4.5 | 16.5 | 4.5 |  |
| 4 or more | 16.2 | 11.9 | 18.6 | 18.1 | 20.0 | 9.4 | 12.7 | 9.2 | .506 |
| **Occupational care** |  |  |  |  |  |  |  |  |  |
| Occupational physician | 6.6 | 3.8 | 11.6 | 4.6 | 11.1 | 3.4 | 1.3 | 2.0 | .083 |
| **Mental healthcare** |  |  |  |  |  |  |  |  |  |
| Psychologist/psychiatrist | 8.4 | 8.2 | 4.7 | 13.0 | 20.0 | 6.6 | 3.8 | 10.0 | .257 |
| **Allied health professionals** |  |  |  |  |  |  |  |  |  |
| Physiotherapist | 26.5 | 12.2 | 32.6 | 10.6 | 20.0 | 19.9 | 26.6 | 9.9 | .673 |
| Speech therapist | 0.6 | 10.0 | 0 | - | 2.2 | 10.0 | 0 | - | .262 |
| Dietician | 6.6 | 2.3 | 0 | - | 4.4 | 2.5 | 11.4 | 2.2 | .081 |
| Occupational therapist | 0 | - | 0 | - | 0 | - | 0 | - | - |
| Total number of different allied health professionals |  |  |  |  |  |  |  |  |  |
| 0 | 64.7 | - | 60.5 | - | 68.9 | - | 64.6 | - |  |
| 1 | 29.9 | 9.2 | 34.9 | 9.9 | 26.7 | 13.5 | 29.1 | 6.6 |  |
| 2 | 4.2 | 17.4 | 4.7 | 12.0 | 2.2 | 21.0 | 5.1 | 19.3 |  |
| 3 | 0.6 | 28.0 | 0 | - | 2.2 | 28.0 | 0 | - |  |
| 4 | 0 | - | 0 | - | 0 | - | 0 | - | .671 |
| **Emergency care** |  |  |  |  |  |  |  |  |  |
| Ambulance rides, N(%), mean | 6.0 | 1.2 | 7.0 | 1.0 | 2.2 | 1.0 | 7.6 | 1.3 | .767 |
| Emergency room visit(s), N(%), mean | 11.4 | 1.3 | 14.0 | 1.3 | 6.7 | 1.0 | 12.7 | 1.3 | .764 |
| Hospital admission(s) N(%), duration | 13.8 | 6.8 | 14.0 | 4.5 | 8.9 | 5.5 | 16.5 | 8.3 | .858 |
| **Home care** |  |  |  |  |  |  |  |  |  |
| Community nurse, N(%), hours | 1.2 | 122.5 | 0 | - | 2.2 | 20.0 | 1.3 | 225.0 | - |
| Informal care, N(%), hours | 3.0 | 87.2 | 4.7 | 118.0 | 2.2 | 20.0 | 2.5 | 90.0 | .315 |
| Household help, N(%), hours | 3.6 | 132.3 | 4.7 | 174.0 | 2.2 | 78.0 | 3.8 | 122.7 | .522 |
| NFPA (non-functioning pituitary adenoma), N (number), SD (standard deviation)  p-value based on number and frequency of visits, (bold) p < 0.05 | | | | | | | | | |
