# Supplementary material for: Healthcare utilization and costs among patients with non-functioning pituitary adenomas
Source: Endocrine. 2019 Mar 22;64(2):330–40. doi: 10.1007/s12020-019-01847-7 (PMC6531397; doi:10.1007/s12020-019-01847-7)
Supplement: Supplementary file 5 — Supplementary Table 3a [file 12020_2019_1847_MOESM5_ESM.docx]

| **Supplementary table 3a.** Medical and medication costs in euros (€) over the past 12 months in 167 patients with an NFPA categorized by treatment strategy | | | | | | | | | | | | | | | | | | |
| --- | --- | --- | --- | --- | --- | --- | --- | --- | --- | --- | --- | --- | --- | --- | --- | --- | --- | --- |
|  | **Total**  **(N=167)** | | | | **Wait-and-scan**  **(N=22)** | | | | **Surgery**  **(N=104)** | | | | **Postoperative radiotherapy**  **(N=41)** | | | | |  |
| ***Medical costs*** | Number of patients | | Costs among those visiting | | Number of patients | | Costs among those visiting | | Number of patients | | Costs among those visiting | | Number of patients | | Costs among those visiting | | | Overall  p-value |
|  | N | % | mean | SD | N | % | mean | SD | N | % | mean | SD | N | % | mean | | SD |  |
| General practitioner | 86 | 51.5 | 135.67 | 131.28 | 9 | 40.9 | 132.0 | 80.8 | 53 | 51.0 | 133.2 | 125.2 | 19 | 46.3 | 144.2 | | 168.3 | .829 |
| Specialist care | 165 | 98.8 | 444.52 | 511.96 | 21 | 95.5 | 485.3 | 393.9 | 103 | 99.0 | 460.3 | 596.2 | 41 | 100.0 | 377.7 | | 293.3 | .636 |
| Allied health professionals* | 58 | 34.9 | 348.21 | 378.54 | 7 | 31.8 | 231 | 117.4 | 30 | 28.8 | 304.7 | 330.9 | 21 | 51.2 | 449.4 | | 476.6 | **.014** |
| Mental healthcare** | 14 | 8.4 | 525.71 | 449.88 | 0 | 0.0 | - | - | 8 | 7.7 | 600.0 | 472.7 | 6 | 14.6 | 426.7 | | 439.1 | .468 |
| Ambulance rides | 10 | 6.0 | 618.00 | 325.71 | 2 | 9.1 | 515.0 | 0 | 5 | 4.8 | 515.0 | 0 | 3 | 7.3 | 858.3 | | 594.7 | .442 |
| Emergency room visits | 19 | 11.4 | 327.16 | 169.22 | 3 | 13.6 | 259.0 | 0 | 10 | 9.6 | 310.8 | 163.8 | 6 | 14.6 | 388.5 | | 216.7 | .466 |
| Hospitalization | 23 | 13.8 | 6188.00 | 10737.13 | 5 | 22.7 | 3427.2 | 2389.5 | 14 | 13.5 | 4692.0 | 7463.1 | 4 | 9.8 | 14875.0 | | 21741.5 | .609 |
| Home care*** | 7 | 4.2 | 12094.57 | 5731.49 | 1 | 4.5 | 4800.0 | - | 5 | 4.8 | 13356.4 | 5808.6 | 1 | 2.4 | 13080 | | - | .690 |
| **Total medical costs** | 167 | 100 | 2103.43 | 6420.38 | 22 | 100.0 | 1670.0 | 2670.1 | 104 | 100.0 | 1986.2 | 4842.8 | 41 | 100.0 | 2633.3 | | 10320.3 | .815 |
| **Medication costs** | Number of patients | | Costs among those using medication | | Number of patients | | Costs among those using medication | | Number of patients | | Costs among those using medication | | Number of patients | | Costs among those using medication | | | Overall  p-value |
|  | N | % | mean | SD | N | % | mean | SD | N | % | mean | SD | N | % | mean | SD | |  |
| Androgel | 56 | 33.5 | 445.88 | 258.61 | 6 | 27.3 | 297.0 | 148.9 | 35 | 33.7 | 427.3 | 243.8 | 15 | 36.6 | 548.8 | 297.8 | | .201 |
| Desmopressine | 11 | 6.6 | 99.12 | 66.92 | 1 | 4.5 | 53.5 | - | 6 | 5.8 | 81.8 | 70.2 | 4 | 9.8 | 136.5 | 59.7 | | .229 |
| Thyrax | 90 | 53.9 | 34.29 | 12.94 | 5 | 22.7 | 35.9 | 10.5 | 55 | 52.9 | 32.6 | 12.5 | 30 | 73.2 | 37.1 | 13.9 | | **.001** |
| Genotropin | 30 | 18.0 | 2917.47 | 1505.46 | 2 | 9.1 | 1407.7 | 1016.8 | 14 | 13.5 | 3233.0 | 1679.7 | 14 | 34.1 | 2817.6 | 1298.6 | | **.025** |
| Cabergoline | 3 | 1.8 | 1887.71 | 2227.07 | 1 | 4.5 | 4423.9 | - | 1 | 1.0 | 251.4 | - | 1 | 2.4 | 987.8 | - | | .052 |
| Quinagolide | 2 | 1.2 | 1056.00 | 1405.73 | 2 | 9.1 | 1056.0 | 1405.7 | 0 | 0.0 | - | - | 0 | 0.0 | - | - | | **.029** |
| Hydrocortison | 77 | 46.1 | 411.82 | 218.94 | 6 | 27.3 | 270.6 | 145.9 | 43 | 41.3 | 435.4 | 268.1 | 28 | 68.3 | 405.9 | 117.4 | | **.007** |
| Anticonceptives | 4 | 2.4 | 43.41 | 31.03 | 2 | 9.1 | 27.9 | 0 | 1 | 1.0 | 90.0 | - | 1 | 2.4 | 27.9 | - | | .629 |
| **Total drug costs** | 125 | 74.9 | 1250.63 | 1610.18 | 13 | 59.1 | 1003.5 | 1439.7 | 76 | 73.1 | 1073.2 | 1554.3 | 36 | 87.8 | 1714.4 | 1727.9 | | **.016** |
| **Overall costs** | 167 | 100 | 3039.53 | 6498.22 | 22 | 100.0 | 2263.0 | 3119.9 | 104 | 100.0 | 2770.5 | 4932.2 | 41 | 100.0 | 4138.6 | 10282.6 | | .437 |
| NFPA (non-functioning pituitary adenoma), N (number), IQR (interquartile range), (bold) p < 0.05  * Physiotherapists, Speech therapists, Dieticians, Occupational therapists  ** Psychiatrists, psychologists  ***Community nurse, informal care, household helpReference prices are presented in supplementary table 4 | | | | | | | | | | | | | | | | | | |
