# Supplementary material for: Healthcare utilization and costs among patients with non-functioning pituitary adenomas
Source: Endocrine. 2019 Mar 22;64(2):330–40. doi: 10.1007/s12020-019-01847-7 (PMC6531397; doi:10.1007/s12020-019-01847-7)
Supplement: Supplementary file 6 — Supplementary Table 3b [file 12020_2019_1847_MOESM6_ESM.docx]

| **Supplementary table 3b.** Medical and medication costs in euros (€) over the past 12 months in 167 patients with an NFPA categorized by follow-up | | | | | | | | | | | | | | | | | |
| --- | --- | --- | --- | --- | --- | --- | --- | --- | --- | --- | --- | --- | --- | --- | --- | --- | --- |
|  | **Total**  **(N=167)** | | | | **0-5 years**  **(N=43)** | | | | **5-10 years**  **(N=45)** | | | | **>10 years**  **(N=79)** | | | |  |
| **Medical costs** | Number of patients | | Costs among those visiting | | Number of patients | | Costs among those using medication | | Number of patients | | Costs among those using medication | | Number of patients | | Costs among those using medication | | Overall  p-value |
|  | N | % | mean | SD | N | % | mean | SD | N | % | mean | SD | N | % | mean | SD |  |
| General practitioner | 86 | 51.5 | 135.67 | 131.28 | 24 | 55.8 | 141.6 | 133.2 | 18 | 40.0 | 186.8 | 44.0 | 39 | 49.4 | 117.6 | 95.7 | .829 |
| Specialist care | 165 | 98.8 | 444.52 | 511.96 | 42 | 97.7 | 630.5 | 849.0 | 45 | 100.0 | 444.9 | 338.4 | 78 | 98.7 | 344.2 | 282.7 | .636 |
| Allied health professionals* | 58 | 34.9 | 348.21 | 378.54 | 17 | 39.5 | 333.9 | 213.8 | 14 | 31.1 | 497.4 | 513.3 | 27 | 34.2 | 279.9 | 370.6 | **.014** |
| Mental healthcare** | 14 | 8.4 | 525.71 | 449.88 | 2 | 4.7 | 832.0 | 633.6 | 9 | 20.0 | 419.6 | 389.4 | 3 | 3.8 | 640.0 | 586.6 | .468 |
| Ambulance rides | 10 | 6.0 | 618.00 | 325.71 | 3 | 7.0 | 515.0 | 0 | 1 | 2.2 | 515.0 | - | 6 | 7.6 | 686.7 | 420.5 | .442 |
| Emergency room visits | 19 | 11.4 | 327.16 | 169.22 | 6 | 14.0 | 345.3 | 211.5 | 3 | 6.7 | 259.0 | 0 | 10 | 12.7 | 336.7 | 174.8 | .466 |
| Hospitalization | 23 | 13.8 | 6188.00 | 10737.13 | 6 | 14.0 | 5950.0 | 11121.5 | 4 | 8.9 | 3570.0 | 2734.4 | 13 | 16.5 | 7103.4 | 12439.1 | .609 |
| Home care*** | 7 | 4.2 | 12094.57 | 5731.49 | 2 | 4.7 | 18602.0 | 828.7 | 1 | 2.2 | 7094.0 | - | 4 | 5.1 | 10091.0 | 4853.1 | .690 |
| **Total medical costs** | 167 | 100 | 2103.43 | 6420.38 | 43 | 100.0 | 2645.1 | 6641.6 | 45 | 100.0 | 1254.0 | 1893.4 | 79 | 100.0 | 2292.5 | 7831.9 | .815 |
| **Medication costs** | Number of patients | | Costs among those using medication | | Number of patients | | Costs among those using medication | | Number of patients | | Costs among those using medication | | Number of patients | | Costs among those using medication | | Overall  p-value |
|  | N | % | mean | SD | N | % | mean | SD | N | % | mean | SD | N | % | mean | SD |  |
| Androgel | 56 | 33.5 | 445.88 | 258.61 | 14 | 32.6 | 429.4 | 199.2 | 14 | 31.1 | 490.0 | 331.5 | 28 | 35.4 | 432.0 | 250.7 | .201 |
| Desmopressine | 11 | 6.6 | 99.12 | 66.92 | 2 | 4.7 | 148.4 | 67.1 | 4 | 8.9 | 101.0 | 67.0 | 5 | 6.3 | 78.0 | 70.2 | .229 |
| Thyrax | 90 | 53.9 | 34.29 | 12.94 | 22 | 51.2 | 29.7 | 13.7 | 20 | 44.4 | 34.7 | 11.1 | 48 | 60.8 | 36.3 | 13.0 | **.001** |
| Genotropin | 30 | 18.0 | 2917.47 | 1505.46 | 4 | 9.3 | 2905.0 | 898.9 | 8 | 17.8 | 2293.6 | 889.9 | 18 | 22.8 | 3197.5 | 1769.6 | **.025** |
| Cabergoline | 3 | 1.8 | 1887.71 | 2227.07 | 1 | 2.3 | 4423.9 | - | 1 | 2.2 | 251.4 | - | 1 | 1.3 | 987.8 | - | .052 |
| Quinagolide | 2 | 1.2 | 1056.00 | 1405.73 | 1 | 2.3 | 62.0 | - | 1 | 2.2 | 2050.0 | - | 0 | 0.0 | - | - | **.029** |
| Hydrocortison | 77 | 46.1 | 411.82 | 218.94 | 13 | 30.2 | 429.4 | 394.3 | 15 | 33.3 | 443.5 | 246.2 | 49 | 62.0 | 397.5 | 136.9 | **.007** |
| Anticonceptives | 4 | 2.4 | 43.41 | 31.03 | 0 | 0.0 | - | - | 2 | 4.4 | 27.9 | 0 | 2 | 2.5 | 58.9 | 43.9 | .629 |
| **Total drug costs** | 125 | 74.9 | 1250.63 | 1610.18 | 31 | 72.1 | 922.2 | 1248.4 | 29 | 64.4 | 1147.1 | 1458.4 | 65 | 82.3 | 1453.5 | 1806.5 | **.016** |
| **Overall costs** | 167 | 100 | 3039.53 | 6498.22 | 43 | 100.0 | 3310.0 | 6578.8 | 45 | 100.0 | 1993.2 | 2162.7 | 79 | 100.0 | 3488.3 | 7940.3 | .437 |
| NFPA (non-functioning pituitary adenoma), N (number), IQR (interquartile range), (bold) p < 0.05  * Physiotherapists, Speech therapists, Dieticians, Occupational therapists  ** Psychiatrists, psychologists  ***Community nurse, informal care, household help  Reference prices are presented in supplementary table 4 | | | | | | | | | | | | | | | | | |
