# Supplementary material for: Healthcare utilization and costs among patients with non-functioning pituitary adenomas
Source: Endocrine. 2019 Mar 22;64(2):330–40. doi: 10.1007/s12020-019-01847-7 (PMC6531397; doi:10.1007/s12020-019-01847-7)
Supplement: Supplementary file 7 — Supplementary Table 4 [file 12020_2019_1847_MOESM7_ESM.docx]

| **Supplementary table 4.** Unit costs in euros (€) | | | | |
| --- | --- | --- | --- | --- |
| ***Direct medical costs*** | ***Value (€)*** | ***Reference*** | ***Source*** | ***Remark*** |
| General practitioner | 33 | 2016 | Guideline* | Per visit |
| Specialist care | 91 | 2016 | Guideline* | Per visit |
| Paramedical care** | 33 | 2016 | Guideline* | Per visit |
| Mental healthcare*** | 64-98 | 2016 | Guideline* | Per visit |
| Ambulance rides | 515 | 2016 | Guideline* | Per visit |
| Emergency room visits | 259 | 2016 | Guideline* | Per visit |
| Inpatient care | 476 | 2016 | Guideline* | Per visit |
| Community nurse | 73 | 2016 | Guideline* | Per visit |
| Informal care | 50 | 2016 | Guideline* | Per visit |
| Household help | 20 | 2016 | Guideline* | Per visit |
| **Drug costs** |  |  |  |  |
| Androgel | 0.31-10.49 | 2018 | Medicijnkosten.nl | Dependent on individual dosage, price per unit, plus 6€ for a prescription |
| Desmopressine | 0.13-1.00 | 2018 | Medicijnkosten.nl | Dependent on individual dosage, price per unit, plus 6€ for a prescription |
| Thyrax | 0.02-0.32 | 2018 | Medicijnkosten.nl | Dependent on individual dosage, price per unit, plus 6€ for a prescription |
| Genotropin | 3.34-10.13 | 2018 | Medicijnkosten.nl | Dependent on individual dosage, price per unit, plus 6€ for a prescription |
| Cabergoline | 4.72 | 2018 | Medicijnkosten.nl | Dependent on individual dosage, price per unit, plus 6€ for a prescription |
| Quinagolide | 0.80 | 2018 | Medicijnkosten.nl | Dependent on individual dosage, price per unit, plus 6€ for a prescription |
| Hydrocortison | 0.02-2.50 | 2018 | Medicijnkosten.nl | Dependent on individual dosage, price per unit, plus 6€ for a prescription |
| Anticonceptives | 0.06-2.20 | 2018 | Medicijnkosten.nl | Dependent on individual dosage, price per unit, plus 6€ for a prescription |
| * Dutch guidelines for healthcare cost calculation  ** Physiotherapists, Speech therapists, Dieticians, Occupational therapists  *** Psychiatrists, psychologists | | | | |
