# Supplementary material for: Healthcare utilization and costs among patients with non-functioning pituitary adenomas
Source: Endocrine. 2019 Mar 22;64(2):330–40. doi: 10.1007/s12020-019-01847-7 (PMC6531397; doi:10.1007/s12020-019-01847-7)
Supplement: Supplementary file 8 — Supplementary Table 5a [file 12020_2019_1847_MOESM8_ESM.docx]

| **Supplementary table 5a.** Disease bother and needs for support among 167 patients with a non-functioning adenoma categorized by endocrine deficits corrected for age and gender | | | | | | | |
| --- | --- | --- | --- | --- | --- | --- | --- |
|  | **Total**  **(N=167)** | | **No deficits**  **(N=46)*** | | **Hypopituitarism**  **(N=121)*** | | **P-value*** |
| **Disease bother** | mean | SD | mean | SD | mean | SD |  |
| Physical & cognitive complaints | 16.9 | 20.0 | 12.5 | 19.2 | 19.0 | 19.6 | .058 |
| Mood | 14.1 | 19.1 | 11.9 | 19.0 | 15.0 | 18.5 | .372 |
| Negative illness perceptions | 11.5 | 15.7 | 11.7 | 15.6 | 11.4 | 15.3 | .914 |
| Sexual functioning | 14.8 | 21.1 | 9.4 | 20.5 | 16.7 | 20.6 | **.048** |
| Social functioning | 7.9 | 16.1 | 5.4 | 15.7 | 9.1 | 16.4 | .192 |
| Total index score | 13.4 | 15.9 | 10.7 | 15.2 | 14.5 | 15.1 | .181 |
| **Needs for support** |  |  |  |  |  |  |  |
| Physical & cognitive complaints | 17.8 | 23.1 | 10.7 | 22.1 | 21.1 | 21.8 | **.007** |
| Mood | 16.4 | 23.7 | 11.3 | 23.2 | 18.6 | 22.9 | .078 |
| Negative illness perceptions | 17.5 | 23.5 | 16.3 | 23.2 | 18.0 | 23.0 | .682 |
| Sexual functioning | 14.5 | 23.0 | 8.2 | 22.0 | 17.0 | 22.7 | **.028** |
| Social functioning | 8.7 | 18.5 | 4.2 | 17.9 | 10.8 | 18.6 | **.041** |
| Total index score | 15.4 | 19.6 | 10.2 | 18.6 | 17.7 | 19.4 | **.030** |
| NFPA (non-functioning pituitary adenoma), N (number), SD (standard deviation), (bold) p<0.05  Lower scores indicate lower disease bother and lower needs  * corrected for age and gender | | | | | | | |
