# Supplementary material for: Healthcare utilization and costs among patients with non-functioning pituitary adenomas
Source: Endocrine. 2019 Mar 22;64(2):330–40. doi: 10.1007/s12020-019-01847-7 (PMC6531397; doi:10.1007/s12020-019-01847-7)
Supplement: Supplementary file 9 — Supplementary Table 5b [file 12020_2019_1847_MOESM9_ESM.docx]

| **Supplementary table 5b.** Disease bother and needs for support among 167 patients with a non-functioning adenoma categorized by endocrine deficits corrected for age and gender | | | | | | | |
| --- | --- | --- | --- | --- | --- | --- | --- |
|  | **Wait-and-scan**  **(N=22)*** | | **Surgery**  **(N=104)*** | | **Radiotherapy**  **(N=41)*** | | **P-value*** |
| **Disease bother** | mean | SD | mean | SD | mean | SD |  |
| Physical & cognitive complaints | 12.2 | 18.8 | 16.2 | 19.0 | 22.1 | 18.6 | .099 |
| Mood | 13.3 | 18.8 | 13.0 | 19.0 | 17.2 | 19.0 | .482 |
| Negative illness perceptions | 9.5 | 15.1 | 9.8 | 15.1 | 16.6 | 15.2 | **.047** |
| Sexual functioning | 17.1 | 20.2 | 12.8 | 19.8 | 18.2 | 20.0 | .314 |
| Social functioning | 6.3 | 15.9 | 7.7 | 16.1 | 10.1 | 15.8 | .602 |
| Total index score | 11.9 | 15.2 | 12.2 | 14.8 | 17.5 | 15.0 | .165 |
| **Needs for support** |  |  |  |  |  |  |  |
| Physical & cognitive complaints | 11.7 | 22.0 | 17.3 | 22.1 | 23.5 | 21.8 | .110 |
| Mood | 12.9 | 23.0 | 15.7 | 23.1 | 20.4 | 23.4 | .411 |
| Negative illness perceptions | 11.6 | 22.5 | 15.5 | 22.3 | 25.7 | 22.1 | **.024** |
| Sexual functioning | 16.3 | 22.0 | 12.0 | 21.9 | 20.2 | 21.9 | .127 |
| Social functioning | 5.7 | 17.8 | 7.4 | 18.2 | 14.7 | 17.7 | .061 |
| Total index score | 11.7 | 18.8 | 14.0 | 18.8 | 21.8 | 18.7 | .053 |
| NFPA (non-functioning pituitary adenoma), N (number), SD (standard deviation), (bold) p<0.05  Lower scores indicate lower disease bother and lower needs  * corrected for age and gender | | | | | | | |
