# Supplementary material for: Healthcare utilization and costs among patients with non-functioning pituitary adenomas
Source: Endocrine. 2019 Mar 22;64(2):330–40. doi: 10.1007/s12020-019-01847-7 (PMC6531397; doi:10.1007/s12020-019-01847-7)
Supplement: Supplementary file 10 — Supplementary Table 5c [file 12020_2019_1847_MOESM10_ESM.docx]

| **Supplementary table 5c.** Disease bother and needs for support among 167 patients with a non-functioning adenoma categorized by follow-up corrected for age and gender | | | | | | | |
| --- | --- | --- | --- | --- | --- | --- | --- |
|  | **0-5 years**  **(N=43)*** | | **5-10 years**  **(N=45)*** | | **>10 years**  **(N=79)*** | | **P-value*** |
| **Disease bother** | mean | SD | mean | SD | mean | SD |  |
| Physical & cognitive complaints | 12.6 | 18.8 | 23.6 | 18.8 | 15.9 | 19.2 | **.015** |
| Mood | 11.9 | 18.8 | 18.5 | 18.8 | 12.7 | 19.1 | .175 |
| Negative illness perceptions | 9.7 | 15.6 | 14.6 | 15.4 | 10.5 | 15.6 | .250 |
| Sexual functioning | 15.2 | 20.5 | 13.9 | 20.1 | 15.0 | 20.4 | .947 |
| Social functioning | 6.0 | 15.7 | 11.4 | 15.4 | 7.3 | 15.6 | .229 |
| Total index score | 11.0 | 15.6 | 17.4 | 15.4 | 12.4 | 15.3 | .105 |
| **Needs for support** |  |  |  |  |  |  |  |
| Physical & cognitive complaints | 14.5 | 22.0 | 24.2 | 22.1 | 16.6 | 21.9 | .081 |
| Mood | 16.6 | 23.3 | 19.8 | 23.5 | 14.5 | 23.5 | .489 |
| Negative illness perceptions | 14.8 | 23.3 | 21.3 | 22.8 | 16.8 | 22.8 | .380 |
| Sexual functioning | 13.9 | 22.4 | 15.4 | 22.1 | 14.4 | 22.2 | .949 |
| Social functioning | 6.9 | 18.4 | 11.1 | 18.1 | 8.8 | 18.3 | .551 |
| Total index score | 13.7 | 19.4 | 19.4 | 18.8 | 14.5 | 18.8 | .295 |
| NFPA (non-functioning pituitary adenoma), N (number), SD (standard deviation), (bold) p<0.05  Lower scores indicate lower disease bother and lower needs  * corrected for age and gender | | | | | | | |
